# Daily Life Stress and the Cortisol Awakening Response: Testing the Anticipation Hypothesis

**DOI:** 10.1371/journal.pone.0052067

**Published:** 2012-12-20

**Authors:** Daniel J. Powell, Wolff Schlotz

**Affiliations:** 1 Faculty of Social and Human Sciences, University of Southampton, Southampton, United Kingdom; 2 Institute of Psychology, University of Regensburg, Regensburg, Germany; Max Planck Institute for Evolutionary Anthropology, Germany

## Abstract

The cortisol awakening response (CAR) is a distinct facet of the circadian cortisol rhythm associated with various health conditions and risk factors. It has repeatedly been suggested that the CAR could be a result of the anticipated demands of the upcoming day (stress anticipation) and could support coping with daily life stress. In a sample of 23 healthy participants CARs were assessed on two consecutive days by measures of salivary cortisol upon awakening (S1) and 30 and 45 minutes later, which were aggregated to the area under the curve increase (AUCI). Stress anticipation was assessed immediately after awakening. On the same days, daily life stress and distress were assessed six times per day based on a quasi-randomized design using handheld computers. Associations were tested by day using regression analysis and standard multilevel/mixed effects models for longitudinal data. The CAR AUCI moderated the effect of daily life stress on distress; higher CAR increases were associated with attenuated distress responses to daily life stress on both days (day 1: *p* = .039; day 2: *p* = .004) adjusted for age, gender, sleep quality, time of awakening and oral contraceptive use. Lagged-effects and redundancy models showed that this effect was not due to prior-day CAR increases but specific for same day CARs. On day 2, associations between daily life stress and distress were stronger when individuals showed a higher S1 cortisol level, but this effect was similar for S1 on day 1, and the day 2 effect of S1 became non-significant when S1 on day 1 was controlled. No associations were found between stress anticipation and CARs. Findings indicate that the CAR increase is associated with successful coping with same-day daily life stress.

## Introduction

The cortisol awakening response (CAR) is a distinct facet of the circadian cortisol rhythm, an increase of cortisol within the first hour after awakening that is separate from the cortisol increase during the second half of the night [1]. It has been suggested that the CAR is the result of an interaction of hypothalamus-pituitary-adrenal (HPA) axis activity, regional brain activation, and changes in adrenal sensitivity around the process of awakening [2]. Since its initial systematic description [3] the CAR has received considerable research interest. Studies found evidence for associations with a variety of psychosocial factors [4] as well as physical and mental disorders and associated risk factors. For example, increased CARs were observed in relapsing-remitting multiple sclerosis [5], upper respiratory symptoms [6], visceral obesity [7], and women with the metabolic syndrome [8]. In contrast, decreased CARs were observed in patients with type 2 diabetes mellitus [9], chronic fatigue syndrome [10], [11], systemic hypertension [12], and functional gastrointestinal disorders [13]. Clarifying underlying factors and potential consequences of the CAR might help to better understand its often ambiguous links with a variety of health conditions. Two recent longitudinal studies found that a greater CAR is a risk factor for peritraumatic dissociation and acute stress disorder [14], and for major depression [15].

Since the earliest observations of associations of the CAR with psychosocial factors, researchers have repeatedly speculated about functions of the CAR. Uncovering functions of the CAR would serve two goals. First, it might provide an explanation why the CAR has been preserved, and, second, it might help to elucidate biopsychosocial mechanisms in health conditions associated with dysregulation of the CAR. Probably due to the position of the CAR at the beginning of the human activity phase, a recurrent theme has emphasized the potential role of the CAR in dealing with daily life demands within the upcoming day. In the following, this is referred to as the *CAR anticipation hypothesis*. Despite various reiterations of this hypothesis and some largely circumstantial evidence, a systematic test is still outstanding. In the following we will review history and varieties of the CAR anticipation hypothesis, provide a summary of the core claims, and provide tests of some selected deductions.

### Origins and Development of the CAR Anticipation Hypothesis

On the basis of associations between the CAR and chronic stress in an early study by Schulz and colleagues [16] it was speculated that the CAR might serve the specific function of preparing the organism for coping with the demands of the upcoming day: “increased levels of cortisol in the morning might reflect an enhanced need for energy to meet demands. Availability of glucocorticoids promotes a multitude of physiological functions and leads to a state of enhanced arousal” (p. 96) [16], and further: “It is likely that individuals with an excessive number of duties and tasks already engage in the process of coping with these duties as soon as they wake up in the morning” (p. 95). Schulz and colleagues suggested that this process would trigger a stress response in addition to the circadian cortisol increase.

This hypothesis was taken up in a later publication [17] and further developed on the basis of findings of associations of the CAR with chronic stress as well as weekend-weekday differences in the CAR. The authors suggested that the CAR might reflect anticipation effects of upcoming everyday demands, where cognitive preoccupation with upcoming tasks act as strong cognitive or internal stressors potentially linked to CAR increases [17]. However, it has been suggested that expectations upon awakening might not coincide with actual experiences [18]. This theoretical account of the CAR emphasizes its adaptive role in that it provides the individual with energy needed to meet the anticipated demands of the upcoming day [19].

Wilhelm and colleagues suggested that this process might be based on a neuronal mechanism associated with awakening [1], particularly activation of neocortical networks by brain stem systems, and that this neocortical activation results in reactivation of memory representations that might eventually stimulate the HPA axis [1]. In their review of CAR-related evidence, Fries and colleagues later summarized that “the cortisol rise after awakening may accompany an activation of prospective memory representations at awakening enabling individual’s orientation about the self in time and space as well as anticipation of demands of the upcoming day.” (p. 71) [20] They also emphasized the role of the hippocampus, and that this hypothesis would be consistent with findings of attenuated CARs in patients with hippocampal damage [21], [22].

### Summary of the CAR Anticipation Hypothesis

The key points of these contributions emphasize that the CAR (1) helps to prepare the organism for demands of the upcoming day [16], [17], [19]; (2) is adaptive in that it supports coping with upcoming demands [17], [19]; (3) is linked to anticipation of these upcoming demands [17], [18], [19]; (4) is linked to the reactivation of memory representations via activation of neocortical networks, thereby stimulating HPA axis activity [1], [20]; (5) is associated with individual preconscious and/or conscious internal and external information [16], [17], [18]. A summary of these considerations yields the following CAR anticipation hypotheses:

The CAR is a distinct facet of the circadian rhythm of cortisol secretion that occurs after awakening, is linked to reactivation of information from memory based on neuronal activation processes throughout the awakening period, and serves the function of preparing the organism to deal with demands of the upcoming day. The CAR therefore is adaptive (e.g. supports coping with daily demands) and is linked to anticipatory processes that may or may not be conscious.

This hypothesis is consistent with the evidence from CAR studies presented above. Nevertheless, it must be emphasized that most of this evidence is indirect or circumstantial. Few studies provided evidence that is more directly relevant to the CAR anticipation hypothesis. First, the observation of an increased CAR on competition days compared to non-competition days in competitive ballroom dancers [23] is consistent with the proposed function of preparing the organism to cope with demands of the upcoming day, although it remains unclear if the increased CAR led to better performance and/or activated more performance-relevant resources. Second, findings of an increased CAR on weekdays compared to weekend days [17], [18] similarly support this proposed function in the context of a standard work schedule, although this finding needs to be replicated using objective assessments of sleep and compliance [24]. Third, a single-case study found evidence for an increased CAR if high demands were anticipated for the upcoming day [25], but generalizability to other people is uncertain.

Based on the previously discussed research we tested the following hypotheses: (1) Early morning anticipation of stress on the upcoming day (stress anticipation) is associated with the CAR increase on the same day; (2) A higher CAR increase is associated with (2a) more daily life stress and (2b) attenuated distress responses to daily life stress on the same day. To test these hypotheses we used an ambulatory assessment design assessing the CAR, stress anticipation and momentary daily life stress and distress on two consecutive days.

## Methods

### Participants

Participants were recruited using opportunity sampling within the region of Hampshire, England. The study was advertised using posters and an online recruitment tool. Student participants were compensated for their efforts with research course credits. Participants had to be aged 18 to 40 years to be included. Exclusion criteria were (a) any chronic or acute illness and (b) taking any prescribed drugs except oral contraceptives, as verified by self-report. In total, 25 eligible individuals volunteered. One participant was excluded due to consistently non-normal high cortisol measures; another due to completely missing cortisol measures. Thus the final sample consisted of 23 participants ranging in age from 20 to 37 years (*M* = 24.9; *SD = *3.5). Fifteen (65%) were female, of which five (33%) were taking oral contraceptives. Three (13%) were undergraduate students, eight (35%) postgraduate students, and 12 (52%) were not students. None of the students were in an examination phase while taking part in the study.

All participants gave written informed consent. The study was approved by the Ethics Committee of the School of Psychology at the University of Southampton.

### Design

We implemented an ecological momentary assessment design [26] in order to assess experience as it happens, thus capturing dynamic variation, ensuring ecological validity, and reducing recall bias. Assessments were done on two consecutive weekdays; assessment timing was based on two designs. First, an event-related design for the assessments after awakening and, second, a time-based variable occasion design with stratified random sampling for the assessments on the remainder of the day (cf. [27]).

#### Handheld computer

HP iPAQ 111 handheld computers were programmed to study specifications with customized software written for the Windows Mobile 6 operating system. One handheld was specifically programmed as a demonstration tool for training participants during the briefing session. Handhelds gave an acoustic alarm until the participant engaged with the handheld. If not initiated before, the handheld gave an acoustic alarm at 0830 h to waken the participant. Additional alarms were given 30 min and 45 min after initiation to assist the participants with the exact timing of cortisol assessments to capture the CAR, and at six occasions randomly placed within six strata of 1 h 45 min each between 1000 h and 2030 h. During the day (but not during the CAR assessments) an alarm could be postponed by 5–15 min by the participant. Exact times of responses were recorded by the handheld, and time of awakening was defined as the time of the first assessment.

### Measures

#### Cortisol

Cortisol was assessed from saliva collected by the participants using the Cortisol Salivette (Sarstedt, Leicester, UK). Saliva was collected immediately after awakening, and 30 min and 45 min later to capture the CAR. Participants were asked to refrain from eating, brushing their teeth, smoking, engaging in any physical exercise, and to drink only water. Other than these restrictions, participants were free to undergo their usual morning routine. To ensure compliance, during each saliva collection instruction the handheld briefly presented a random three-digit code which the participant recorded on the label of the salivette tube they were using (cf. [28]). Samples with missing or incorrect codes were excluded from the study. The compliance rate was 93%, with 128 usable cortisol samples out of 138 possible.

#### Cortisol Awakening Response (CAR)

Two markers of the CAR were used: First, cortisol measures were aggregated to one indicator, *Area Under the Curve Increase* (AUCI; [29]), an indicator of cortisol change over time. Second, the cortisol level upon awakening (S1) was used as a marker of the cortisol rise pre-awakening [2].

#### Stress anticipation

Anticipation of stress on the upcoming day (stress anticipation) was assessed by the Anticipatory Stress Questionnaire (ASQ), which was developed for this study [30]. The ASQ was presented by the handheld computer immediately after awakening, and responses were given on an 11-point scale (0 = ‘disagree’; 10 = ‘agree’). Part-whole corrected item-test correlations (i.e. correlations between individual scores on one item and the score on the test which the item is a part of without the contribution of the specific item) were used to evaluate the assumption of homogeneity of the scale. Due to relatively low item-test correlations of 1 of the initial set of 6 items, this item was removed and the remaining 5 items showed acceptable part-whole corrected item-test correlations (.51 ≤ *r*_it_ ≤.87 on both days), with Cronbach’s α = .87 on day 1 and α = .90 on day 2 (Cronbach’s α is an estimator of a scale’s internal consistency [31], [32], with values >.80 indicating good internal consistency by convention, and values >.60 acceptable for group studies and short scales). Item wording was: (1) ‘I expect the upcoming day to be a stressful experience’; (2) ‘I feel in control of those events expected to occur today’; (3) ‘I am confident I can cope with what challenges today presents’; (4) ‘I feel adequately prepared for the upcoming day’; (5) ‘I am worried about how today may turn out’.

#### Sleep quality

Sleep quality was assessed by a single item that was presented after awakening: ‘How would you rate your sleep quality?’ (0 = ‘very bad’; 10 = ‘very good’).

#### Momentary self-reports of stressors and affect

A total of 8 stressor items and 15 negative affect adjectives were presented upon an acoustic alarm given by the handheld. Each item had a response slider (0 = ‘not at all’ to 10 = ‘very much so’) and a ‘not applicable’ option resulting in a missing value. Stressor items were headed by ‘Since the last signal’ to capture all stressors occurring throughout the day. Affect items were headed by ‘At the moment I feel’ to assess momentary affect. To find groups of items that might reflect broader constructs, responses to stressor and negative affect items were subjected to two-level exploratory factor analyses (assessments nested within subjects) for categorical outcomes [33] because of non-normally distributed response variables. Responses were recoded into five categories with cut-offs 1, 3, 6, and 8. A good model fit is indicated by a non-significant χ^2^-value or a χ^2^/df ratio<2; CFI and TLI>.95 and RMSEA<.06, with models closely approaching these values being acceptable [34].

The two-level exploratory factor analysis of the *momentary stressor ratings* resulted in a three-factor solution (Eigenvalues: 2.62; 1.32; 1.04) with good model fit (χ^2^ = 7.1; *df* = 7; *p* = .42; comparative fit index (CFI) = 1.0; Tucker-Lewis index (TLI) = 0.99; root mean square error of approximation (RMSEA) = 0.007) with a satisfactory simple structure after Geomin rotation. The first factor comprised 4 items (‘I performed some of my tasks inadequately’; ‘Others undervalued my work’; ‘I felt discontented with the type of work I'm doing’; ‘I had a disagreement with someone’), reflecting *daily life stress* from negative social evaluation of task performance. Items loading on this factor were used to form the scale Daily Life Stress. The scale showed acceptable internal consistency as indicated by the first assessment of the day (Cronbach’s α: day 1 α = .67; day 2 α = .62; note that due to non-normality of the response variable distributions, alphas are likely to be underestimated and less accurate [35]). Due to technical reasons, the total number of available observations within participants (*m*) dropped slightly on the second day (day 1: *m* = 132 (96% of 138 possible); day 2: *m* = 111 (80%)).

The two-level exploratory factor analysis of the *momentary negative affect ratings* also resulted in a three-factor solution (Eigenvalues: 5.79; 2.16; 1.40) with acceptable model fit (χ^2^ = 94.2; *df* = 63; *p* = .01; CFI = 0.98; TLI = 0.94; RMSEA = 0.043) and a satisfactory simple structure after Geomin rotation. Eight items loaded on the first factor (‘Distressed’; ‘Upset’; ‘Irritable’; ‘Anxious’; ‘Satisfied’ [r]; ‘Calm’ [r]; ‘Down’; ‘Worried’; ‘Angry’, where [r] indicates reverse scored items) reflecting *momentary distress*. Items loading on this factor were used to form the scale Momentary Distress. The scale showed good internal consistency as indicated by the first assessment of the day (Cronbach’s α: day 1 α = .89; day 2 α = .89; note that due to non-normality of the response variable distributions, alphas are likely to be underestimated and less accurate [35]). The total number of available observations was *m* = 133 (96% of 138 possible) on day 1 and *m* = 115 (83%) on day 2.

### Procedure

On the day prior to commencing the two-day protocol, participants reported to the laboratory for a session lasting approximately thirty minutes. On arrival, the study was explained to participants and written informed consent was obtained. Detailed training was then given regarding using and labeling salivettes and operating the handheld computers. The session ended with participants receiving a handheld computer, two bags (one for each sampling day) containing salivettes and detailed instruction sheets pertaining to the two-day protocol. Participants started the assessment on the next day for two consecutive weekdays and returned the salivettes and handheld to the laboratory when finished.

Immediately after awakening, participants initiated the handheld, took a saliva sample, labeled the salivette tube, and answered the ASQ and sleep quality questions. At assessment 30 min and 45 min later, only saliva samples were collected and labeled. Finally, participants completed assessments of stressors and negative affect 6 times during the day. This procedure was repeated on the next day. Participants were instructed to store the saliva samples in the fridge until they returned them to the laboratory.

### Biochemical Analysis

After saliva samples were returned to the laboratory they were stored at −20°C until they were shipped to the Biochemical Lab at the Division of Theoretical and Clinical Psychobiology, University of Trier, Germany, where they were analyzed using a time-resolved immunoassay with fluorescence detection [36]. All samples were measured in duplicate with an average intra-assay coefficient of variation (CV) of 3.8%. Inter-assay CVs were <10%.

### Statistical Analysis

Initially, correlations of cortisol, sleep quality, awakening time, stress anticipation, and average levels of daily life stress and distress were computed. Variable means were compared between days using *t*-tests to check that days were not significantly different and could be used as replication samples.

As the CAR is characterized by both, cortisol level at awakening (S1) and cortisol increase within the first 45 minutes after awakening (AUCI), in the following analysis each model was computed twice, one for AUCI and the other for S1.

#### Tests of hypothesis 1

To test our first hypothesis, a set of four ordinary least squares regression models was constructed. Model 1 (same-day model) tested associations between ASQ (predictor) scores and the CAR AUCI, and S1, respectively, as outcomes, using regression models without covariates. Model 2 (same-day model adjusted) was adjusted for potentially influential covariates: time of awakening, sleep quality, oral contraceptive use, age and gender. Model 3 (lagged-effect model) tested specificity of associations using lagged stress anticipation on day 1 as predictor of AUCI and S1, respectively, on day 2. The purpose of this model was to test if any effect of day-2 anticipatory stress was specific to that day, or if anticipatory stress on the other day had a similar association, which would argue against our hypothesis which assumes day-specificity of effects. Finally, model 4 (redundancy model) tested the relative contribution of anticipatory stress on days 1 and 2 by including both same-day and lagged ASQ scores. If a day-specific effect of anticipatory stress existed in models 1 and 2 but disappeared in model 4, the hypothesis of day-specificity would not be supported. In contrast, the original effect seen in Models 1 and 2 might be due to either person-level anticipatory stress difference (if anticipatory stress on both days would contribute similarly to the outcome, i.e. partial redundancy) or if only the association of day-1-anticipatory stress remained (full redundancy of anticipatory stress on day 2).

In addition, to test if stress anticipation predicted actual stress experienced on the same day (as indicated by momentary stress ratings), the mean of daily life stress within subjects and days was computed. As this variable showed a positively skewed distribution that could not be transformed to normality, four categories with approximately equal numbers of observations were generated and an ordered logit regression model [37] was used to predict the ordinal daily life stress variable from ASQ scores for each day.

#### Tests of hypothesis 2

To test our second hypothesis (does the CAR predict daily life stress and distress responses to stress?) we ran two sets of models. To test hypothesis 2a, ordered logit regression models were used to predict the ordinal daily life stress variable (described above) from AUCI and S1 for each day.

Second, to test for a potential effect of the CAR on distress responses to stress exposure (i.e. hypothesis 2b), we ran a set of models that followed the same analytical logic as the regression models described for testing hypothesis 1. To account for non-independence of repeated measures within individuals and missing observations in our data, we used statistical models that account for the nested structure of observations in persons. Depending on the field of research, different terms have been used to describe such models, e.g. random-effects model for longitudinal data [38], random-coefficient multilevel model [39], mixed-effects regression model [40], or hierarchical linear model [41]. Common characteristics among these models for our specific application are that they include a random effect of time to account for non-independence of observations within subjects, and that they use maximum likelihood estimation to estimate parameters and variance components. In the following, we describe the models we tested for the predictor AUCI (just replace with S1 to get models with S1 as predictor) using the notation of Singer and Willet [42].

Model 1 (same-day model) tested associations of CAR AUCI or S1, respectively, momentary daily life stress rating, and their interaction with momentary distress, with no covariates included.
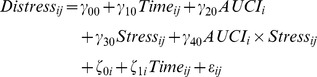



Where *Distress_ij_* is the value of momentary distress for person *i* at measurement occasion (time) *j*, *Time_ij_* the time of day (centered at 1000 h) for person *i* at measurement occasion *j*, *AUCI_i_* the value of AUCI (centered at the sample mean within day) for person *i*, and *Stress_ij_* the value of daily life stress (centered at the person mean within day) for person *i* at measurement occasion *j*. Fixed effects are denoted by γ, with γ_00_ denoting the average intercept, γ_10_ the average effect of Time, γ_20_ the average effect of AUCI, γ_30_ the average effect of daily life stress, and γ_40_ the interaction effect of AUCI with daily life stress. Random effects are denoted by ζ, with ζ_0*i*_ indicating the deviation of the intercept of person *i* from the average intercept, and ζ_1*i*_ the deviation of the effect of person *i* from the average effect of time. Finally, ε*_ij_* denotes residuals at the level of individual observations. The following assumptions about distributions of random effect parameters and residuals were made:
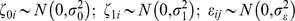



All predictors and outcomes were measured on the same day, and the model was similarly applied to day 1 and day 2. The parameter of primary interest describes the interaction between AUCI and daily life stress (γ_40_). Significance of this parameter indicates that the association of daily life stress with momentary distress is dependent upon the value of AUCI (or S1, respectively, in the model testing the effect of the cortisol level at awakening).

Model 2 (same-day model adjusted) was adjusted for potentially influential person-level covariates (time of awakening, sleep quality, use of oral contraceptives, age and gender).

Model 3 (lagged-effect model) tested lagged effects of the day 1 AUCI and S1, respectively, on day 2 momentary distress. As our hypothesis assumes day-specificity of CAR effects, these tests should result in non-significant AUCI/S1 and interaction effects.

Finally, model 4 (redundancy model) tested effects of predictors on day 2 in the context of predictors on day 1 by including both day 2 and day 1 AUCI and S1, respectively, and their interaction with day 2 daily life stress to predict day 2 momentary distress. Parameters of predictors on day 2 should remain significant as the hypothesis assumes day-specificity.

#### Statistical software and assumption checks

Two-level exploratory factor analyses of momentary stressor and negative affect ratings were done using Mplus 6.11 (Muthén & Muthén, 1998–2011, Los Angeles, CA, USA). All other analyses were done using Stata 12.1 (StataCorp, 1985–2012, College Station, TX, USA). We used an alpha level of p≤.05 for all statistical tests. Assumptions of all statistical models used were checked by testing for potentially influential outliers; potential collinearity of predictors; linearity of associations; normality of residuals (in mixed models also normality of random effects); and homogeneity of error variances. There were no substantial deviations, thus indicating appropriateness of the models.

## Results

### CAR, Stress, Distress, and Sleep by Day

Table 1 shows means of major variables on the two study days. Cortisol levels increased after awakening on average by approximately 3 nmol/L (Day 1) and 5 nmol/L (Day 2) but did not significantly differ between days. Sleep quality, awakening time and stress anticipation also did not significantly differ between days (Table 1). The momentary non-aggregated daily life stress ratings ranged from 0 to 8, the distress ratings from 0 to 8.3, thus reflecting good variability and a sufficient degree of stress and distress experience in this sample. The means of their aggregates also did not significantly differ between day 1 and day 2 (Table 1) and thus were suitable for replicating findings of one day on the other.

**Table 1:** Summary statistics (Mean and *SD*) by day for cortisol measures immediately after awakening (0 min), 30 and 45 min later, an aggregate measure of the cortisol awakening response, sleep quality, awakening time and momentary daily life stress and distress aggregated over the day.

Variable	Day 1			Day 2			t-test
	*M*	*SD*	Min; Max	*M*	*SD*	Min; Max	
Cortisol 0 min (nmol/L)	10.4	6.1	2.2; 24.5	8.5	3.9	1.8; 15.7	*t(19) = 1.5, p* = .15
Cortisol +30 min (nmol/L)	13.4	4.9	5.0; 21.5	13.8	5.1	5.2; 23.1	*t(19) = 0.2, p* = .86
Cortisol +45 min (nmol/L)	13.5	5.9	6.4; 28.5	13.1	6.3	4.2; 31.1	*t(19) = 0.3, p* = .30
CAR AUCI	99.0	211.0	−262; 551	153.7	186.9	−128; 660	*t(19) = *−*1.1, p* = .29
Sleep quality	5.3	2.8	0; 9	5.6	2.7	0; 9	*t(21) = *−*0.8, p* = .43
Awakening time (h since midnight)	7.7	0.9	6.1; 8.6	7.6	0.8	6.1; 8.6	*t(21) = 1.1, p* = .30
ASQ (stress anticipation)	5.7	0.9	4.4; 7.2	6.7	0.9	4.4; 8.0	*t(21) = 0.3, p* = .79
Daily life stress (aggregate)	1.9	0.9	0.3; 3.3	2.1	1.3	0.3; 4.7	*t(22) = *−*0.7, p* = .51
Momentary distress (aggregate)	2.9	1.3	0.8; 5.6	3.1	1.4	0.7; 5.2	*t(22) = *−*0.5, p* = .66

*Note*. CAR AUCI = cortisol awakening response area under the curve increase; ASQ = Anticipatory Stress Questionnaire.

Difference tests are t-tests for paired samples.

Table 2 shows correlations between the main variables. Stability of the variables as shown in the diagonal was moderate, thus supporting the assumption of day-to-day variability. Associations between variables within days were modest, with the exception of expected associations between cortisol at awakening and the CAR AUCI, and between daily life stress and distress.

**Table 2:** Spearman correlations of main variables by study day (*n* = 20–23).

Variable	Cortisol 0 min (S1)	CAR AUCI	Awakening time	Sleep quality	ASQ	Daily life stress (aggregate)	Daily life distress (aggregate)
Cortisol 0 min (S1)	.48*	−.70***	−.05	.01	.13	.35	.18
CAR AUCI	−.55**	.41	.32	.07	.24	−.27	−.06
Awakening time	−.26	.09	.47*	−.27	.38	−.10	.09
Sleep quality	−.25	.61**	−.04	.79***	−.19	.16	.20
ASQ	.05	.14	−.11	.12	.35	.28	.12
Daily life stress (aggregate)	.30	−.02	−.04	.07	.30	.56**	.69***
Daily life distress (aggregate)	.10	−.08	−.03	−.17	−.02	.49*	.52*

*Note*. CAR AUCI = cortisol awakening response area under the curve increase; ASQ = Anticipatory Stress Questionnaire.

+ *p* ≤.10;

* *p* ≤.05;

** *p* ≤.01;

*** *p* ≤.001.

Day 1 above diagonal, day 2 below diagonal. Diagonal shows stability of variables across days.

### Hypothesis 1: Stress Anticipation and CAR

We hypothesized that early morning anticipation of stress on the upcoming day as measured by the ASQ would be associated with CAR increase on the same day. Regression model 1 showed no significant same-day association between ASQ scores and the CAR AUCI on day 1 (standardized regression coefficient: *β* = .18; unstandardized coefficient: *b* = 46.8; *SE* = 58.8; *p* = .44) or day 2 (*β* = .03; *b* = 5.3; *SE* = 47.1; *p* = .91). Similarly, there was no association between ASQ scores and the cortisol level immediately after awakening (S1; day 1: *β* = .13; *b* = 0.9; *SE* = 1.7; *p* = .59; day 2: *β* = .03; *b* = 0.1; *SE* = 1.0; *p* = .89). Results were the same when the models were adjusted for relevant covariates (Model 2; outcome AUCI: day 1: *β* = .16; *b* = 39.4; *SE* = 59.8; *p* = .52; day 2: *β* = .03; *b* = 5.2; *SE* = 40.2; *p* = .90; outcome S1; day 1: *β* = .26; *b* = 1.9; *SE* = 1.9; *p* = .33; day 2: *β* = .04; *b* = 0.2; *SE* = 1.0; *p* = .86). For AUCI, the lagged effects model (Model 3) and the redundancy model (Model 4) showed no significant association, while there was a positive association of cortisol levels immediately after awakening (S1) on day 1 (but not on day 2) with ASQ scores on day 2 (Model 3: *β* = .52; *b* = 2.4; *SE* = 1.05; *p* = .037), which remained significant when day 2 S1 was included in the model (Model 4: *β* = .66; *b* = 3.1; *SE* = 1.2; *p* = .028).

ASQ scores did not significantly predict average daily life stress on day 1 (*coefficient* = 0.51; *SE* = 0.45; *p* = .26) or day 2 (*coefficient* = 0.66; *SE* = 0.44; *p* = .14). Similarly, ASQ scores did not predict daily life distress (both *p*s>.57).

### Hypothesis 2: CAR, Stress and Distress on the Upcoming Day

#### Hypothesis 2a

Based on the assumption that the CAR is an adaptive physiological process that supports coping with demands of the upcoming day, our first prediction was that higher CAR increases might be associated with more daily life stress. The regression model with daily life stress categories as outcome adjusted for age, gender, sleep quality, oral contraceptive use and time of awakening showed trends towards negative associations of CAR AUCI with average daily life stress on both days (day 1: *coefficient* = −0.005; *SE* = 0.003; *p* = .10; day 2: *coefficient* = −0.005; *SE* = 0.003; *p* = .12). In contrast, higher S1 was associated with *higher* average life stress ratings with a trend on day 1 (*coefficient* = 0.159; *SE* = 0.092; *p* = .084), but statistically significant on day 2 (*coefficient* = 0.252; *SE* = 0.127; *p* = .048).

#### Hypothesis 2b

Next we tested if the CAR influences distress responses to daily life stress using the two CAR indicators, S1 and AUCI. Results for S1 are shown in Table 3. The interaction effect in Model 1 shows a trend towards increased distress responses in individuals with relatively high S1 cortisol levels on day 1, which was statistically significant on day 2; these results did not change when adjusting the model for potential confounders (Model 2). Results of Model 3 show that this effect was not specific to the assessment day, with the interaction of daily life stress with S1 *on day 1* significantly predicting distress responses *on day 2*. When controlling for same-day S1 and its interaction with daily life stress, this effect clearly failed to reach significance (Model 4), thus suggesting that the interaction effects were partially redundant and not specific to the day.

**Table 3:** Results of models predicting momentary distress from the cortisol level at awakening (S1) and momentary daily life stress.

	Day 1			Day 2		
	Coef.	*SE*	*p*	Coef.	*SE*	*p*
*Model 1: Same-day model*						
S1 (same day)	0.023	0.049	.64	0.026	0.069	.71
Daily Life Stress (same day)	0.549	0.107	<.001	0.515	0.066	<.001
S1 (same day)×Daily LifeStress (same day)	0.021	0.013	.095	0.048	0.021	.026
*Model 2: Same-day model* *adjusted* a						
S1 (same day)	0.018	0.049	.72	0.039	0.070	.58
Daily Life Stress (same day)	0.547	0.107	<.001	0.525	0.067	<.001
S1 (same day)×Daily LifeStress (same day)	0.021	0.013	.093	0.050	0.021	.020
*Model 3: Lagged-effect model* a						
S1 (day 1)				0.087	0.039	.025
Daily Life Stress (day 2)				0.554	0.080	<.001
S1 (day 1)×Daily Life Stress(day 2)				0.029	0.011	.005
*Model 4: Redundancy model* a						
S1 (day 2)				0.068	0.067	.31
S1 (day 1)				0.058	0.043	.18
Daily Life Stress (day 2)				0.562	0.082	<.001
S1 (day 2)×Daily Life Stress(day 2)				0.031	0.025	.23
S1 (day 1)×Daily Life Stress(day 2)				0.018	0.013	.18

*Note*. Effects of covariates and fixed and random effects of intercept and time not shown. S1 = cortisol level immediately after awakening.

a Model adjusted for same-day awakening time, same-day sleep quality, age and gender.

**Figure 1:**
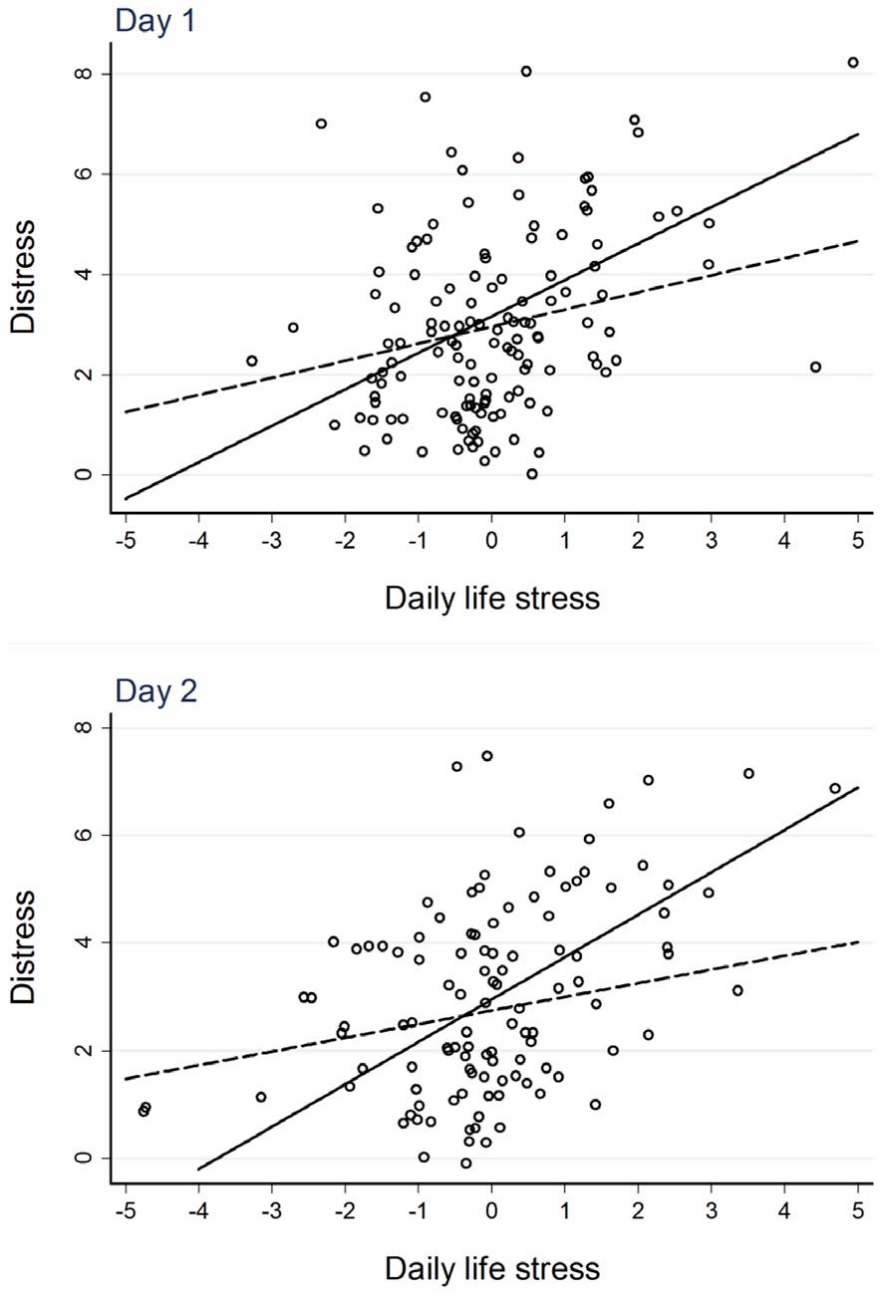
Illustration of the attenuation of distress responses to daily life stress (within-person centered) by the cortisol awakening response (CAR) increase (as indicated by the area under the curve increase, AUCI, see text for details) on study days 1 and 2. Solid lines show the association at 1 standard deviation (*SD*) below average CAR AUCI (i.e. relatively low CAR increase), dashed lines that at 1 *SD* above average CAR AUCI (i.e. relatively high CAR increase). On both days, distress was found to be lower at relatively high levels of daily life stress if the cortisol awakening response was high, whereas no differences in distress were seen at within-person average levels of daily life stress.

Table 4 shows the results for the cortisol *increase* after awakening (AUCI). Model 1 yielded two main results. First, daily life stress had a strong positive main effect on distress, thus demonstrating the stress-distress association in daily life at the sample average of AUCI. Second, the interaction term with CAR AUCI was significant and negative on both days, meaning that the stress-distress association was *attenuated* in individuals with a higher CAR increase. Figure 1 illustrates this effect. The effects were stable when adjusting the model for covariates (Table 4, Model 2). In contrast, results of the lagged-effect model showed that the AUCI on day 1 did not significantly attenuate the stress-distress association on day 2 (non-significant interaction parameter in Table 4, Model 3). Finally testing the redundancy by including day 1 and day 2 AUCIs in the model showed that the day-specific interaction effect of AUCI with daily life stress to attenuate momentary distress remained significant (Table 4, Model 4).

**Table 4:** Results of models predicting momentary distress from the cortisol awakening response increase (AUCI) and momentary daily life stress.

	Day 1			Day 2		
	Coef.	*SE*	*p*	Coef.	*SE*	*p*
*Model 1: Same-day model*						
AUCI (same day)	−0.0005	0.0014	.72	−0.0007	0.0014	.61
Daily Life Stress (same day)	0.5138	0.1092	<.001	0.5546	0.0671	<.001
AUCI (same day)×Daily Life Stress (same day)	−0.0009	0.0005	.044	−0.0016	0.0005	.003
*Model 2: Same-day model adjusted* a						
AUCI (same day)	−0.0011	0.0016	.51	−0.0009	0.0017	.60
Daily Life Stress (same day)	0.5097	0.1086	<.001	0.5640	0.0673	<.001
AUCI (same day)×Daily Life Stress (same day)	−0.0010	0.0005	.039	−0.0015	0.0005	.004
*Model 3: Lagged-effect model* a						
AUCI (day 1)				−0.0022	0.0015	.14
Daily Life Stress (day 2)				0.5933	0.0759	<.001
AUCI (day 1)×Daily Life Stress (day 2)				−0.0004	0.0003	.19
*Model 4: Redundancy model* a						
AUCI (day 2)				−0.0009	0.0015	.57
AUCI (day 1)				−0.0020	0.0015	.18
Daily Life Stress (day 2)				0.6131	0.0747	<.001
AUCI (day 2)×Daily Life Stress (day 2)				−0.0014	0.0007	.042
AUCI (day 1)×Daily Life Stress (day 2)				0.0001	0.0004	.84

*Note*. Effects of covariates and fixed and random effects of intercept and time not shown. AUCI = cortisol awakening response area under the curve increase.

a Model adjusted for same-day awakening time, same-day sleep quality, oral contraceptive use, age and gender.

## Discussion

We hypothesized that (1) the CAR would be associated with stress anticipation after awakening and (2) a higher CAR would be associated with (2a) more daily life stress and (2b) attenuated responses to these stress events. We found (1) no significant association of the CAR with anticipatory stress on the same day, (2a) mixed evidence for the CAR predicting daily life stress, and (2b) significant moderation effects of the association between daily life stress and distress by the CAR. Whereas higher cortisol levels upon awakening (S1) were associated with stronger distress responses on day 2, we found that a higher cortisol *increase* after awakening (AUCI) clearly *attenuated* distress responses to daily life stress, and this effect was evident on both study days. This effect is consistent with our second hypothesis, it was remarkably consistent across days, and it remained significant when the models were adjusted for age, gender, time of awakening, oral contraceptives, and sleep quality. It is important to note that the interaction of daily life stress with cortisol *increases* remained significant when adjusting for prior-day cortisol increases, whereas the effect of awakening levels disappeared, thus indicating a carry-over effect of prior-day awakening levels. This is consistent with findings of state-trait analyses [43] where it was found that the cortisol *increase* after awakening (indicated by the AUCI) is much more strongly influenced by state factors than the mean level after awakening (indicated here by S1).

Overall, these results support our hypothesis that strong CAR *increases* are associated with reduced distress resulting from daily life stress. Further support is provided by the observation that this effect was specific to the same day, which means that it was not resulting from a third variable on the person-level.

In contrast, we did not find supporting evidence for our first hypothesis, as there were no significant associations between stress anticipation and same-day CARs. Although the regression coefficients were in the predicted direction on both days, they clearly failed to reach statistical significance. Three potential reasons should be considered. First, statistical power was limited due to the relatively small sample. Post-hoc power analysis [44] showed that our test was adequately powered (*power* = .85) to detect a large effect (*r* = .50). Therefore, a larger sample would be needed to detect a smaller effect if it exists. Second, the stress anticipation processes relevant for triggering CAR increases might not be conscious, as earlier suggested by Kunz-Ebrecht and colleagues [18]. Stress anticipation was not significantly associated with mean daily life stress as assessed on a momentary basis, and the amount of shared variance was modest (correlations of aggregates were *r* = .28 on day 1 and *r* = .30 on day 2 as shown in Table 2). Thus, self-reports of anticipatory stress reflect only part of the actual stress experience on the upcoming day, which would support the speculation that part of the stress anticipation process is not available to conscious information processing [1]. Third, the ASQ assessment directly after awakening might not have captured all relevant anticipations of upcoming demands to be reflected in the cortisol assessments 30 or 45 minutes after awakening. Ideally, stress anticipation should be assessed repeatedly up to ∼40 minutes after awakening, as a cortisol response can be mounted in ∼10 minutes.

In line with our prediction of higher levels of daily life stress on days with a higher CAR (hypothesis 2a) we found associations of higher S1 with more daily life stress on day 2, which failed to reach significance on day 1. However, contrary to our predictions we found trends towards lower levels of daily life stress on days with a higher AUCI. Although these associations were rather weak, this hypothesis should be further explored in future studies. Note that we had a broad sample of momentary stress assessments across the day over two days, so inadequate statistical power or assessment bias are unlikely reasons for the failure to find a significant effect.

We found attenuated distress responses to daily life stress within-subjects if the person showed a relatively high cortisol increase after awakening (hypothesis 2b). It can be speculated that this attenuation effect on distress responses to stress indicates an adaptive function of the CAR and might explain why it has been preserved. Cortisol is known to interact with physiological processes to increase energy availability for coping with demands, which might include permissive and preparative actions of morning cortisol levels [45], [46]. With regards to distress, a number of studies showed that stress-related cortisol increases were associated with lower later levels of distress (e.g., [47], [48], [49]). Therefore, higher cortisol awakening responses might have a protective function, buffering experiences of distress after stress or supporting a quicker return of negative affect to its set-point. Interestingly, a recent neuroimaging study demonstrated reduced responsiveness of the amygdala to negative stimuli as a slow response to exogenous administration of cortisol due to altered coupling of the amygdala with the medial prefrontal cortex [50]. The authors suggested that this slow effect of cortisol helps to prevent overshoot of amygdala activity during stress and enables adequate recovery after stress [50]. These processes would provide a potential mechanism for a slow mood-buffering effect of the CAR covering the rest of the day.

Although reverse effects, i.e. effects of stress or distress on the CAR earlier on the same day, can be eliminated due to the time lag between CAR and daily life stress assessments, it needs to be emphasized that underlying mechanisms of this association are unknown. Alternative to the above mentioned potentially relevant interactions of cortisol with amygdala responsiveness to negative stimuli, the observed associations might be due to a third variable affecting both, CAR and distress responses. However, results of the redundancy model showed that a relevant variable would need to show day-level variability, rather than person-level variability (e.g. a personality trait, or chronic adverse environmental conditions). While awakening time and subjective sleep quality were ruled out in our covariate-adjusted models, the roles of other day-level characteristics such as objective sleep indicators or prior-day states such as worrying/rumination [51], loneliness, sadness and stress [19], and positive affect [25] should be investigated in future studies.

A major strength of our study is the replication on a second day, thus strongly limiting the probability of a chance finding. Also, our redundancy model demonstrated the significance of day-to-day changes of the CAR rather than trait-like characteristics. This is consistent with a recent finding that CAR flexibility is more consistently linked with psychosocial factors than trait-like characteristics of the CAR [52]. In this context it is tempting to speculate that some health conditions might be characterized by a failure to mount an adequate CAR on stressful days compared to non-stressful days, as recently shown for surviving cancer patients [53].

Of course, our study has a number of limitations apart from the rather low number of participants. First, sampling more days would be desirable to test lagged effects repeatedly. Second, we had no objective verification of awakening time; finally, physiological stress responses such as cardiovascular activity or endocrine measures such as salivary cortisol [27] should be assessed in synchrony with distress measures to investigate if effects are generalizable to other facets of the stress response. For example, it has been shown that higher levels of the inflammatory marker IL-6 were associated with daily life stress [54] and a less pronounced CAR [55].

In summary, our results suggest that stronger CAR increases are associated with attenuated distress responses to daily life stress on two consecutive days using an ambulatory assessment design. Future studies should try to replicate our findings and could test potential clinical implications.
